# Borax and Bromides in Epilepsy

**Published:** 1893-11-11

**Authors:** 


### NEW DRUGS AND PREPARATIONS.

[We should be glad to receive, at our Office, 428, Strand, London, W.C., from the manufacturers, specimens of all new preparations and drugs which may be brought out from time to time.]

### BORAX AND BROMIDES IN EPILEPSY—(*continued*).

There is, of course, a close analogy to the state of a person after a bout of drunkenness, where the absorption of trimethyl diamine gives similar symptoms due to the toxine.

Borax is by no means free from the charge of producing trouble of its own. While it improves the mental condition, and helps to keep in check the epilepsy, it is apt to upset the digestion, and cause great gastric irritation and diarrhœa. Wasting and œdema of the extremities may occur if large doses are given; but, indeed, there seems no advantage in having recourse to them. As much as forty grains three times daily have been given alone for a short time, but we prefer ten to twenty grains with a little bromide as more reliable and lasting in its effects. It should be given two or three hours after meals, well diluted, and after a time the stomach generally gets to tolerate it. It is always well to begin with small doses, and to add a little bismuth or some other gastric sedative.

An erythematous or papulous eruption often gives trouble for a time which subsides if the drug is continued, and for this a little ointment or alkaline baths may be advisable. Finally, we may in rare instances find a condition of alopecia develop, to the great alarm of the patient. Dr. Alexander has observed two or three instances of this, but does not leave off the treatment when it occurs, since a spontaneous new growth of the hair follows after a while.

Gowers speaks of borax as distinctly useful in some cases where bromides fail, but gives no indications where it is likely to succeed. The evidence at present seems to point to its value rather in combination with bromide than alone, and especially where mental depression or irritability, or where minor seizures are present. Here it seems to possess great power for good, and deserves to be more widely used, so far as our experience goes. We have not space to say much of nitro-glycerine, which has been occasionally found to be of use. An injection will sometimes cut short or abort a seizure, but no great results have been obtained by its continued administration. Of late, too, antifebrin phenacetin and similar preparations have been found occasionally of value to replace the bromides. Care is needed to prevent dangerous effects, though these rarely occur. The Therapeutic Committee of the British Medical Association notice the comparative danger of antifebrin, especially in full doses.

We must not be thought to overestimate the importance of drugs. A careful analysis of the symptoms should always be made, and any local irritation or organic lesion must be fully treated. If surgical interference is likely to be beneficial, there is no excuse for leaving the patient to the chances of palliation or slow cure by medicinal treatment. In any case, the patient should be inspired with courage and hope, as Dr. Alexander warns us, and should be urged to persevere with his treatment, even for two or three years after losing his seizures, and should sedulously avoid undue excitement, and as carefully seek constant occupation, and, if possible, have cheerful companionship and regular surroundings.

